# A rational study of transduction mechanisms of different materials for all solid contact-ISEs

**DOI:** 10.1038/s41598-024-55729-8

**Published:** 2024-03-05

**Authors:** Heba M. Hashem, A. B. Abdallah

**Affiliations:** 1 Pharmaceutical Analytical Chemistry Department, Faculty of Pharmacy, Mansoura University, Mansoura, 35516 Egypt; 2 Chemistry Department, Faculty of Science, Mansoura University, Mansoura, 35516 Egypt; 3 Chemistry Department, Faculty of Science, New Mansoura University, New Mansoura, Egypt

**Keywords:** Screen printed, Solid contact ion selective electrodes (SC-ISEs), Multi-walled carbon nanotubes (MWCNTs), Poly aniline (PANi), Ferrocene, Venlafaxine, Chemistry, Nanoscience and technology

## Abstract

The new era of solid contact ion selective electrodes (SC-ISEs) miniaturized design has received an extensive amount of concern. Because it eliminated the requirement for ongoing internal solution composition optimization and created a two-phase system with stronger detection limitations. Herein, the determination of venlafaxine HCl is based on a comparison study between different ion- to electron transduction materials (such as; multiwalled carbon nanotubes (MWCNTs), polyaniline (PANi), and ferrocene) and illustrating their mechanisms in their applied sensors. Their different electrochemical features (such as bulk resistance (Rb**), double-layer capacitance (Cdl), geometric capacitance (Cg), and specific capacitance (Cp)) were evaluated and discussed by using the Electrochemical Impedance Spectroscopy (EIS), Chronopotentiometry (CP), and Cyclic Voltammetry (CV) experiments. The results indicated that each transducer's influence on the proposed sensor's electrochemical characteristics is determined by their unique chemical and physical properties. The electrochemical features vary for different solid contact materials used in transduction mechanisms. The results confirm that the MWCNT sensor revealed the best electrochemical behavior with the potentiometric response of a near-Nernestian slope of 56.1 ± 0.8 mV/decade with detection limits of 3.8 × 10^−6^ mol/L (r^2^ = 0.999) and a low potential drift (∆E/∆t) of 34.6 µV/s. Also, the selectivity study was performed in the presence of different interfering species either in single or complex matrices. This demonstrates excellent selectivity, stability, conductivity, and reliability as a VEN-TPB ion pair sensor for accurately measuring VEN in its various formulations. The proposed method was compared to HPLC reported technique and confirmed no significant difference between them. So, the proposed sensors fulfill their solutions' demand features for VEN appraisal.

## Introduction

Over the past half-century, ion selective electrodes (ISEs) have been used as an analytical technique for the assessment of more than 60 analytes in diverse fields, comprising clinical analysis, environmental analysis, physiology, and process control^1^, classic liquid contact ISEs (LC-ISEs) are considered the most common and popular type of analysis in different laboratories and environmental analyses. Recently, the miniaturization and integration of these types of electrodes have attracted a lot of attention for achieving more applicability and accuracy in different fields. So, the first solid-contact ISEs (SC-ISEs) were developed by Cattrall and Freiser in 1971 without the presence of an internal solution called “coated wire electrodes” (CWEs)^2^, Lately, improvements in the limit of detection have likely been achieved by replacing the conventional design of LC-ISEs, in which there is an internal filling solution of the membrane, with a novel design that contains solid inner contact as SC-ISEs. The new design of SC-ISEs has drawn significant attention due to its giving rise to a two-phase system and resulting in more robust detection limits because of the disappearance of internal solution composition that needs to be optimized periodically.

**Figure 1:**
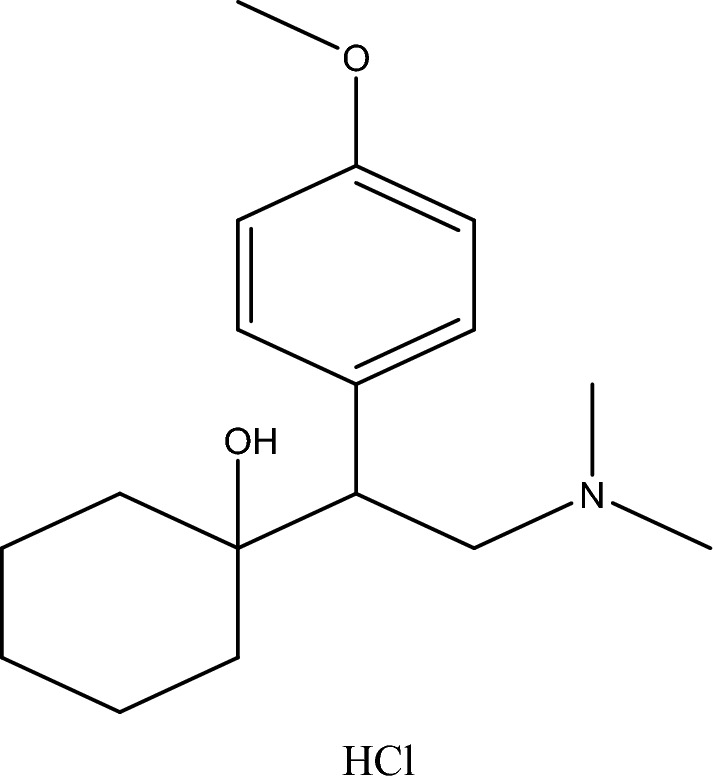
The chemical structure of Venlafaxine hydrochloride.

The essential components of a potentiometric ion-selective membrane are its selective membrane, inner layer (ion-to-electron transduction), and electron-conducting substrate. All boundaries reveal a constant potential difference except the membrane/sample interface; thus, the change in electrode potential depends only on the concentration of the target ion in a sample. As soon as an ion passes through the interface between the organic phase (membrane) and aqueous phase (internal or sample solution), the change in the potential difference that reflects the activities of the target ion is developed. The poor reproducibility and drift potential are considered as the observed limitations exhibited by CWEs. These limitations are backed by the resistance of high charge transfer at the interface between the conductor and the ion-selective membranes (ISM). Thus, it was essential to apply a new electrode design to enhance the transduction and avoid the previous limitations. SC-ISEs were good choices that have a layer between the conductor and the ISM. This layer could act as an ion-to-electron transducer that was first applied by conducting polymers (CP)^3,4^. After that, the successive efforts to enhance the features of the SC-ISEs were achieved by adding different types of solid contact materials such as conducting polymers (polypyrrole^5^, poly(3-octyl thiophene) (POT)^6^, polyaniline (PANi)^7^, and poly(3,4-ethylenedioxythiophene) (PEDOT)^1,8^, single or multiwalled carbon nanotubes (SWCNTs, and MWCNTs), and other materials (e.g., ferrocene). Herein the two mechanisms will briefly illustrate through the phase interfacial potential according to the type of SC material. For example, the solid contact is a conducting polymer or ferrocene with a high redox capacitance. They enhance ion-to-electron transduction because of their electrical and ionic conductance by doping. Poly (3,4-ethylenedioxythiophene) (PEDOT) acts as the template of redox conductive polymers. Through the oxidation or reduction reaction, the desired ion concentration converts to an electron signal. On the other hand, the capacitance of the double layer may be improved by doping the electrode surface by using nanostructured carbon materials^9–11^. Based on that, the cations and anions are formed at ISM, while the other side is formed by electrical charge, i.e., electrons or holes in the SC layer. It is worth mentioning that this approach not only increases the double-layer capacitance but also enhances the interfacial contact area (ISM/ SC) without affecting the geometric projection of the solid contact^12,13^. Under this circumstance, venlafaxine hydrochloride was used as a model drug to investigate the different mechanisms of SC-ISE transduction and proficiency. Venlafaxine hydrochloride (1-[2-(dimethylamino)-1-(4-methoxyphenyl) ethyl] cyclohexanol, VEN, Fig. 1 is considered as one of the non-tricyclic antidepressant drugs that branched from the SNRI category. VEN has an essential and clear effect on treating depression symptoms, panic disorder, social phobia, and generalized anxiety^14^. Also, VEN slightly inhibits dopamine reuptake without significantly harming histaminergic, adrenergic, or muscarinic receptors^15^. Unfortunately, the harmful dose of VEN has exhibited adverse effects seriously, such as cardiac conduction abnormalities, serotonin toxicity, and depression symptoms^14,16^.

Hence, VEN analysis in different matrices has high attention according to a medical and clinical point of view^17^. But most of the reported determination methods of VEN have many limitations, such as long run time, high cost, preliminary preparations, and pre-separation, which lead to their drawbacks in the routine subject analysis. On the other hand, the potentiometric methods based on SC-ISEs offer high applicability and facility in determining various analytes in their matrices without pre-preparations and several extraction steps^9^. The investigation of the reliable and stable potentiometric response of ISEs must be fulfilled with specific features. They have non-polarizable interfaces with high exchange current densities, reversible transitions from ionic to electronic conduction, and vice versa, and the absence of side reactions. For simple and valuable measurement purposes, screen-printed based on ISEs has great attention due to their sensitivity, selectivity, and potential stability^10^. Recently, screen-printed SC- ISEs integrated with polymeric and /or nanomaterials layers have a straightforward approach to monitoring different analytes^18,19^.

Therefore, the main aim of this work is to study the electrochemical performance (e.g., chemical stability, potential drift, capacitance double layer) of different modified SC-ISEs (conductive materials and carbon nanotubes) to detect venlafaxine drug (as an example). Theses layers were characterized with scanning electron microscope (SEM). The performance of these types of SC-ISEs was investigated and compared using potentiometric measurements, electrochemical impedance spectroscopy (EIS), chronopotentiometry (CP), and cyclic voltammetry (CV). The applied sensors were based on PVC plasticized membranes with 2-nitrophenyl octyl ether (*o*-NPOE) and incorporated VEN-TPB^−^ ion-pair, a highly selective and sensitive recognition sensory receptor for VEN determination. The sensors were successfully applied for VEN determination in different dosage forms and synthetic urine samples.

## Materials and methods

### Apparatus

An improved planar chip with a flat printed design, along with double-junction reference electrode Ag/AgCl (Jenway™ 3505) filled with 1 mol/L KCl, were used as the indispensable units for electrical potential measurements represent working and reference electrodes, respectively. Also, the aforementioned electrodes, in conjunction with the bench pH/mV meter (Jenway™ 3510), combined glass pH electrode to conduct all potential measurements and pH measurements. The Electrochemical and Chronopotentiometric measurements were applied by (NOVA 2.0 software; Metrohm Auto lap B.V. Utrecht, The Netherlands). The microscopic morphologies of the modified screen-printed working electrodes were investigated by a scanning electron microscope (SEM, JEOL JSM-6510LV, Oxford instruments, UK) at an acceleration voltage of 30 kV. Millipore Milli-Q system (18.2 MΩ cm specific resistance) was used for de-ionized water suppling. All measurements were exhibited at ambient temperature (25 ± 2 °C).

### Reagents and materials

High molecular weight poly (vinyl chloride) (PVC) and 2-nitrophenyl octyl ether (*o* -NPOE) were purchased from Sigma Aldrich (St. Louis, Mo, USA). Tetrahydrofuran (THF), phosphoric acid, sodium acetate, sodium hydroxide (NaOH), ethanol (EtOH), hydrochloric acid (HCl), potassium chloride, Sodium tetraphenylborate (NaTPB), urea, creatinine, uric acid, citrate, and albuminuria were obtained from Fluka AG (Buchs, Switzerland). MWCNTs were purchased from (EPRI, Cairo, Egypt). Both PANi and ferrocene were purchased from Merck (KGaA, Darmstadt, Germany). Pure VEN powder was supplied kindly from Pharaonia Pharmaceuticals (Alexandria, Egypt). Capsules were purchased from local pharmacies, which were commercially expressed as Effexor XR Extended-Release (Wyeth, United Kingdom) labeled as [75 and 150 mg/capsule], and Venlafaxine Hydrochloride Extended-Release [Auro Pharma Inc, Canada] labeled as 75 mg/capsule.

Different concentrations of VEN (10^−2^–10^−7^ mol/L) were obtained by several dilution of stock solution of 10^–2^ mol/L VEN. The buffer solutions of 10 mmol/L of phosphate buffer were used and were adjusted with aliquots of HCl and/or NaOH. For all EMF measurements, an optimum buffer of pH 6 was used. All solutions and buffers were kept at 4 °C.

### Ion-pair formation

VEN-TPB^−^ ion-pair was prepared by mixing the 10^−2^ mol/L VEN: 10^−1^ mol/L TPB^−^ at the 1:1 ratio. The white precipitate of the ion-pair formed, washed many times with the Millipore Milli-Q system and was centrifuged at 8000 rpm for 1 h. After that, the supernatant was decantated, and then the final powder was dried in an oven under vacuum for 20 min. Finally, the resultant powder was milled to a fine powder in an agate mortar and was stored in a well-sealed bottle. The dried precipitate was subjected to elemental analysis for chemical composition identification.

### Sensor fabrication

**Figure 2:**
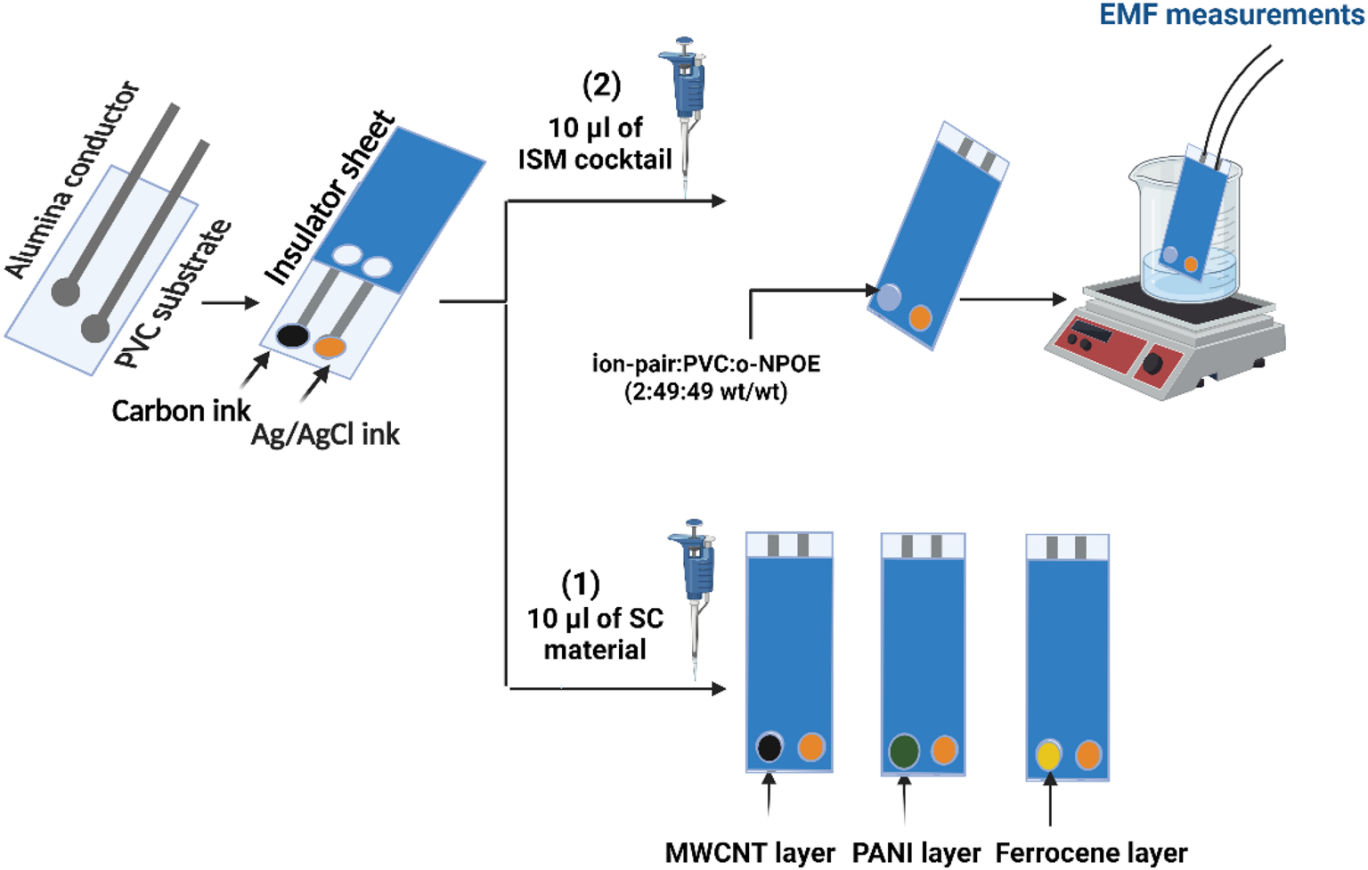
Schematic diagram for fabrication of modified screen-printed electrode.

The printed process was performed manually on a planar screen-printed chip (35 × 0.1 mm) surface. Prior to the printing, the alumina substrate was cleaned several times with EtOH and double distilled water and then dried at room temperature. Then, the carbon layer was deposited on the alumina surface. Subsequently, an insulator layer was spread over the screen-printed surface, solidified by irradiating with ultraviolet and leaving the working area with a conductive track dimension. The two sensing orifices (2 mm width) were printed on a substrate made from carbon ink. The modification of these orifices occurred by using different solid contact materials with the same volume (10-µL of 2 mg/1 mL THF), which were separately drop-casted, such as MWCNT, PANi, or ferrocene. The chip was left to dry completely. All membranes were composed of ion-pair: PVC: *o*-NPOE with a ratio of 2:49:49 wt/wt. All these constituents were dissolved in THF (1.5 mL) and were stirred at 300 rpm for 20 min. Then, 10-µL of the previous membrane were drop-casted on modified sensing orifices and were left to dry as illustrated in (Fig. 2).

EMF measurements were obtained after immersing screen-printed in 10^−2^ mol/L VEN solution for 2 h. EMF measurements plotted a calibration graph versus logarithm [VEN]. The applied sensor was stored in the drug solution when not in use.

### Electrochemical measurements

The electrochemical performance of different modified SC-ISEs was evaluated by EIS, CP, and CV by using one-compartment three-electrode cell using (NOVA 2.0 software; Metrohm Auto lap B.V. Utrecht, The Netherlands) combined with Pt auxiliary electrode and the reference electrode (Ag/AgCl/KCl (3 mol/L). The measurements of EIS and CP were carried out by using a solution of 10^–3^ mol/L of VEN in phosphate buffer (10 mmol/L) of pH 6.0 at ambient temperature 25 ± 1 °C. The spectra of impedance were recorded at the open-circuit potential of 0.01 V with an excitation amplitude of 10 mV. The frequency range starts from 100 kHz to 0.1 Hz^20^.

The capacitive current and specific capacitance of the bare and modified SC-ISEs was also examined by using cyclic voltammetry technique at optimum conditions (potential window; − 0.2 to 0.6 V, scan rate; 100 mV s^−1^) in the electrochemical cell that containing of 25 mL of buffered VEN (1 mmol/L) at pH 6.

### Sensor calibration and selectivity measurements

Calibration graphs were represented as the relation between potential readings and logarithm VEN concentrations. So, all potential readings were obtained by applying some sequence steps as follows. At first, the modified proposed sensor was immersed in a 10-mL beaker contains 10^−2^ mol/L phosphate buffer solution of pH 6.0. Aliquot part (1.0 mL) of standard VEN solution (1.0 × 10^−7^ to 1.0 × 10^−2^ mol/L) were successively added (0.5 by 0.5 mL) with continuous stirring. After that, the potential readings were taken after potential stabilization (10 s). Then the calibration graphs were plotted to exhibit the analytical features of each sensor. Besides, the interferences effect and selectivity coefficients results were investigated by applying the modified separate solution method (MSSM)^21^. Separate calibration curves were plotted for each interference ion of interest, in which the selectivity coefficients were calculated at the highest recorded concentrations (0.1 mol/L).

### Sample preparation

All applications were applied on sensor II of MWCNT-ISE. For pharmaceutical formations and in synthetic urine samples, the VEN stock solution was prepared, the contents of 10 capsules were weighed, and the average weight of the active ingredient per capsule was calculated. The precise amount of powder corresponding to 3.3 g of VEN was then dissolved in 100 mL of deionized water for a VEN stock solution of a 10^–1^ mol/L concentration. The powder was subjected to sonication for 1 h to ensure complete dissolution of the active ingredient. Following sonication, the solution was filtered, and further dilutions were made to obtain various concentrations of VEN ranging from 10^−2^ to 10^−7^ mol/L. Calibration graphs were generated by plotting the potential readings against the logarithm of the VEN concentration, and these graphs were compared to the calibration curves obtained from pure VEN under the same experimental conditions.

The synthetic urea sample was prepared, which contained organic (urea (0.16 M), creatinine (5.2 mM), uric acid (0.991 mM), citrate (1.6 mM), albuminuria, and inorganic species (sodium (0.06 M), potassium (0.02 M), sulfate (0.012 M), calcium (0.001 M), ammonium (0.015 M), phosphate (0.015 M), Magnesium (3 mM), chloride (0.06 M), oxalate (0.18 mM), oxalate (0.012 M)). Later, VEN (5 mM) was added to the prepared urine sample. Subsequently, an appropriate amount of trichloroacetic acid was used to precipitate the protein, and the separation occurred concerning centrifugation at 1500 rpm. After filtration, the concentration of hydrogen in the solution was adjusted to pH 6 using a phosphate buffer.

## Results and discussion

### SEM

**Figure 3:**
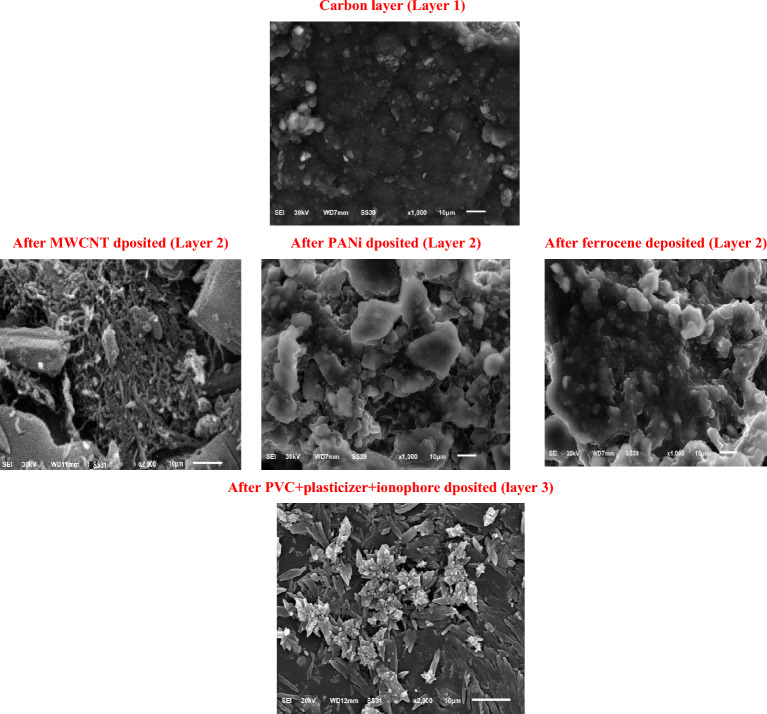
Scanning electron microscope images of different deposited layers at solid electrode surface.

SEM investigated the surface morphologies of the modified SC-ISEs, and the patterns are exhibited in Fig. 3. After the carbon layer was deposited, uniform surface topography was observed. An entangled tubular structure was observed when the MWCNT was dropped onto the carbon layer, which increased the surface area. However, the significant change, porous structure with a uniform distribution, in the SC-ISEs surface morphology was observed after the active polymer (PANi or ferrocene) insertion in the active pore above the carbon layer. In addition, the entangled tubes (MWCNT) and porous structure (PANi and ferrocene) disappeared, and the surface became smooth with edge-shaped needle rods after printing the plasticized polymer containing the ionophore. Overall, the SEM images confirmed the successful printing process at the surface of the SC-ISEs.

### Electrochemical Investigation

The super capacitive performance of the electrode composites can be evaluated via electrochemical methods, namely, electrochemical impedance spectroscopy (EIS), chronopotentiometry (CP), and cyclic Voltammetry (CV).

#### Electrochemical impedance spectroscopy (EIS) measurements

**Figure 4:**
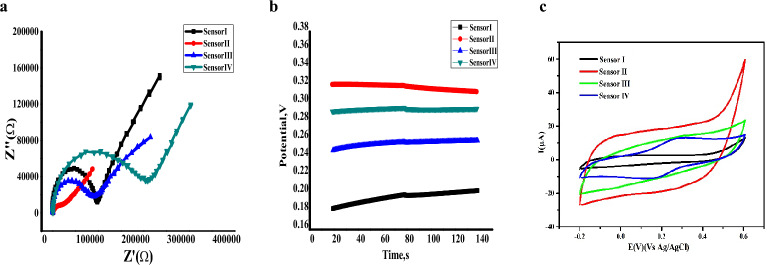
(**a**) Electrochemical impedance spectroscopy (EIS) measurements of the applied sensors in a solution of buffered VEN (1 mM) at frequency range of 0.01–100 kHz at excitation amplitude 100 mV. (**b**) Chronopotentiometry (CP) measurements of the applied sensor buffered VEN (1 mM) (constant current of + 1 nA for 60 s followed by a current of 1 nA for 60 s). (**c**) Cyclic voltammograms (CV) for different ISEs in 25 mL of buffered VEN (1 mmol/L) at pH 6 at a scan rate of 100 mV s^−1^ and potential window is − 0.2–0.6 V.

EIS measurements were carried out to investigate the difference between the electrochemical behaviors of the applied sensors^20^. As shown in Fig. 4A, the method exhibited the Nyquist plots (complex plane plots of − Z^\\^ vs. Z^\^) on the equivalent circuit models. In addition, the bulk resistance (R_b_**), double-layer capacitance (C_dl_), and geometric capacitance (C_g_, in high frequency range) were calculated, and are listed in Table 1.

**Table 1:** EIS, CP and CV features of the proposed sensors.

Parameter	Sensor I	Sensor II	Sensor III	Sensor IV
R_b_** (MΩ)	0.1	0.02	0.09	0.2
C_dl_ (µF)	10.5	33.1	19.0	13.5
C_g_ (nF)	0.68	3.3	0.6	0.88
R_b_* (MΩ)	0.4	0.08	0.3	0.3
∆E/∆t (µV/s)	97.7	34.6	41.6	61.2
C_L_ (µF)	10.2	28.9	24.0	16.3
Cp	0.0038	0.0259	0.0154	0.0108

Comparing the results with four SC-ISEs, the resistance of bulk (Rb**) were 0.1, 0.02, 0.09, and 0.2 MΩ for sensor type I, II, III, and IV, respectively. Rb** values difference revealed the variation in the electron transfer resistance for different modified SC-ISEs based on the change in the semicircle diameter according to the Nyquist diagram. These results reflected that the ferrocene-type sensor had the highest resistance over the normal, which can be attributed to its covalency with the polymeric membrane of the applied sensor. Meanwhile, the MWCNT sensor revealed a minor magnitude of resistance compared to the other types.

In contrast, the measurements of the C_dl_ (Eq. (1)) were conducted on the semicircle low-frequency branch for sensor types I, II, III, and IV are 10.5, 33.1, 19.0, and 13.5 µF, respectively. In this case, the MWCNT sensor presented the highest C_dl_ value owing to the creation of an electrical double layer at the interface between the polymeric ISM and SC layer. Meanwhile, both PANi and ferrocene sensors revealed their C_dl_ values attributable to their transduction mechanisms based on redox reactions, will be illustrated in (3.4. Transduction mechanisms).

1 $$2\pi {C}_{dl}=\frac{d(-Z)}{d(\frac{1}{f})}$$

In which, the imaginary part of the impedance (− Z″) with the reciprocal frequency (1/f) is plotted.

The Cg values were also calculated at high frequencies. MWCNT sensor showed a superb double layer capacitance (33.1 µF) and geometric capacitance (3.3 nF). These results confirm that sensor II, modified with MWCNT, is more effective than the other synthesized sensors.

#### Chronopotentiometry measurements (CP)

Bobacka’s group developed a CP technique for investigation of the potential stability of ISEs^8^. The test preceded under the same conditions as EIS measurements. Figure 4B illustrates the chronopotentiograms, in which each amplitude was applied at current ± 1nA for the 60 s. Furthermore, the potential draft (∆E/∆t), resistance (R_b_*), and capacitance (C_L_) were measured and are listed in Table 3. As expected, sensor II showed low resistance (0.08 MΩ) and potential draft (34.6 µV/s), which emphasized the high stability of the synthesized sensor and exhibited high specific surface areas due to its structure.

The modification of the sensor with an SC transducer investigated the incredibly increasing potential stability. The higher C_L_ magnitude reflected the double layer formation for the MWCNT sensor in good agreement with EIS results.

#### Cyclic voltammetry (CV)

The results are exhibited in Fig. 4C. and from Eq. (2) compared to the specific capacitance (Cp, 0.0038) of bare ISE, Cp of the ISEs after modification with conductive substances (PANi and ferrocene) was dramatically increased as (0.0154, and 0.0108), respectively. Confirming the effectiveness of the ion-to-electron transducers because they are both electrically conductive and exhibit ionic conductivity. While the Cp of MWCNT modification (0.0259) is sharply elevated, that may be due to an electrical double-layer capacitor, which is made up of a carbon‐based supercapacitor. The electrical double-layer is formed by an electrostatic reaction that accumulates positive and negative charges at the interfaces between the electrode and electrolyte, creating an electrical double-layer. The capacitance is the result of the charges building up. There is no electron transport in this charge storage mechanism, and the capacitance is based on the MWCNT’s surface area^22,23^.

2 $${C}_{p}={\int }_{V1}^{V2}\frac{i\left(V\right)d\nu }{\left(V2-V1\right)\nu m}$$

where *i* represents the current response (A), *V* is the voltage (V), *V*2 − *V*1 denotes the potential range (V), *m* indicates the mass of the active materials on the electrode (g), and *v* is the scan rate (V/s).

Conversely, the charge is stored Faradaically in pseudo capacitors, which employ conducting polymers and additional materials like PANi and ferrocene. Redox supercapacitors and pseudo capacitors are more charge-storage capable and have more incredible specific energy. In contrast to electrical double-layer capacitors (EDLCs), they can hold charge in most materials through redox reactions. Nevertheless, they have a lower specific power because the redox reaction process takes longer with pseudo capacitors than with EDLC. High specific energy and power, high specific capacitance, and a long lifespan are necessary for supercapacitors like MWCNT to be called high-performance supercapacitors.

All the previous results of EIS, CP and CV measurements revealed that the impact of each transducer on the electrochemical features of the proposed sensor is according to their chemical and physical natures. The transduction mechanisms for different solid contact materials exhibit different electrochemical features. The results endorse that the MWCNT sensor has high potential stability, conductivity, and high credibility in the VEN-TPB^−^ ion pair sensor for the determination of VEN in its matrices.

### Water layer test (durability)

**Figure 5:**
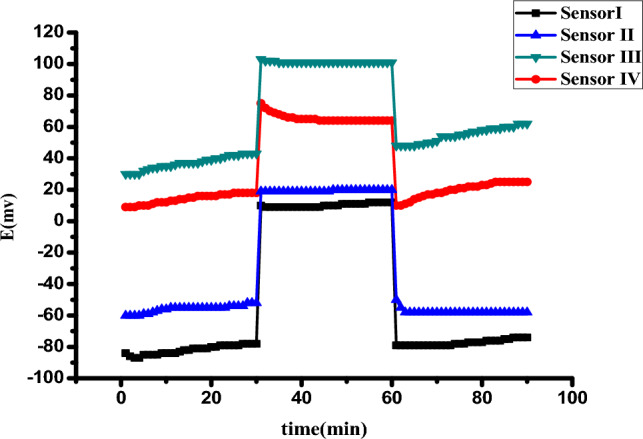
Water-layer tests for the VEN-ISE senor (I) without solid contact, senor (II) MWCNT/ VEN-ISE, sensor (III) PANi / VEN-ISE and sensor (IV) ferrocene/VEN-ISE.

This experiment aimed to evaluate the potential stability and lifespan of the proposed sensor. The test involved immersing the sensors in a solution containing ten mmol/L of phosphate buffer at pH 6, followed by immersion in a 10 mL solution of VEN at a concentration of 10^–4^ mol/L for 30 min. The water layer was formed in the case of the absence of solid contact between ISM and the electronic conductive substrate^24^. As shown in Fig. 5, MWCNT/ VEN-ISE displays the highest potential stability over the operating time of the test. These results are in correlation with CP and EIS measurements.

### Transduction mechanisms

**Figure 6:**
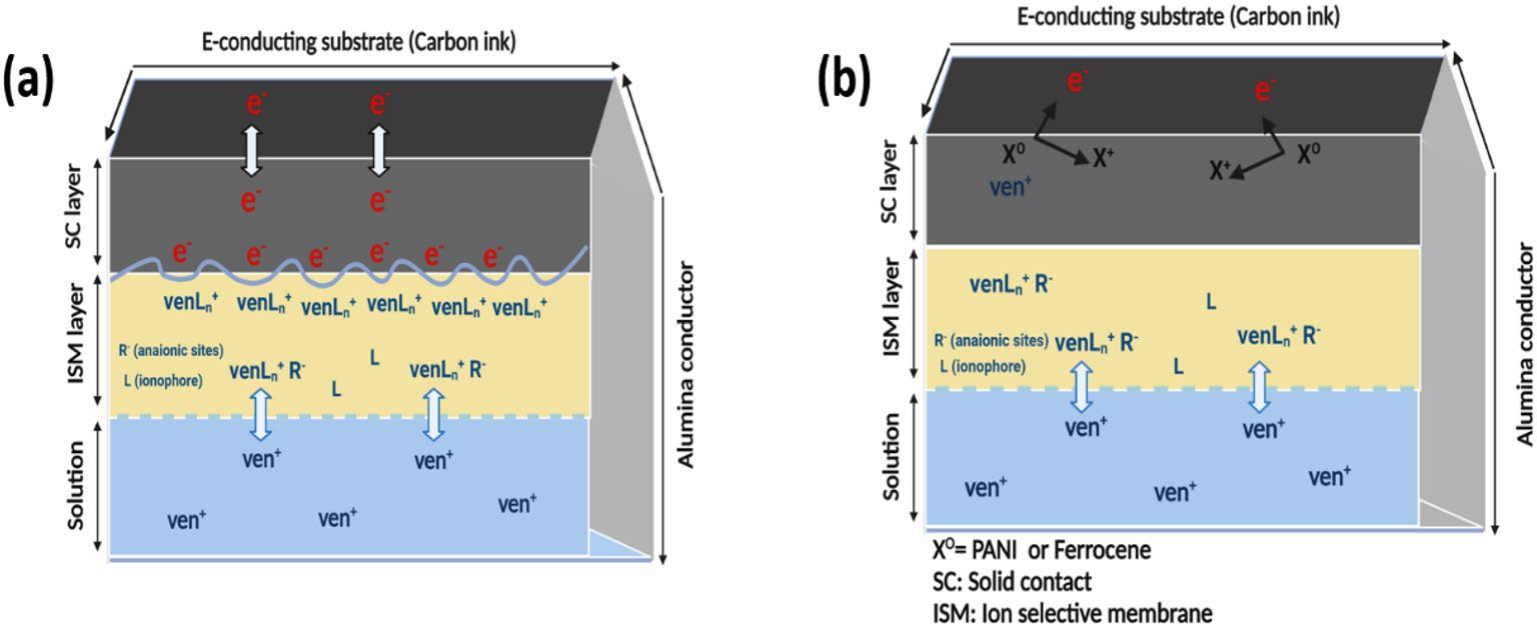
Response mechanisms for the SC-ISEs. (**A**) Electric-double-layer (EDL) capacitance-based SC-ISEs with MWCNT for EDL SC transducer. (**B**) Redox capacitance-based SC-ISEs with PANi and ferrocene for redox SC transducer.

The solid contact materials introduced in this study exhibit two main transduction mechanisms, illustrated in Fig. 6. One of these mechanisms involves multi-walled carbon nanotubes (MWCNT), a nanostructured material with a high surface area. It facilitates ion-to-electron transduction by creating an electrical double layer (EDL) at the interface between the polymeric ion-selective membrane (ISM) and the solid contact (SC) (MWCNT). The EDL is formed by the interaction between cations or anions in the ion-selective membrane and the electrical charge within the porous structure of the MWCNT^25^. The potential at the interface between the electrically conducting substrate and the solid contact is minimal (E ≈ 0). Therefore, solid contact materials based on EDL capacitance are highly electronically conductive. No charge transfer reaction occurs at the interface between the solid contact and the ion-selective membrane. Still, the potential decreases due to the double-layer capacitance, as depicted in Fig. 6A.

Additionally, the transduction mechanisms based on redox reactions can be explored using other electroactive species, such as polyaniline (PANi) and ferrocene, as illustrated in Fig. 6B. These materials serve as redox-active ion-to-electron transducers, where the electrical signals are generated through redox reactions represented by Ox + e^−^  ⇄ Red^1^. The reaction involves three main equilibrium charge transfers at three different boundaries or interfaces. Firstly, at the interface between the electrically conducting substrate and the solid contact (PANi or ferrocene), an electron transfer reaction (ET) of PANi or ferrocene takes place. Secondly, at the interface between the solid contact and the ISM, a charge transfer (CT) occurs due to the ion transfer (IT) of the doped TPB^−^ anion, along with the interfacial potential. Finally, at the interface between the ISM and the aqueous solution, there is a CT equilibrium resulting from the ion transfer of VEN^+^. As a result, the observed potential E confirms the Nernstian response towards the desired ion and highlights the thermodynamic potential of redox capacitance-based solid contact ion-selective electrodes (SC-ISEs).

### Evaluation of the proposed sensors for VEN detection

**Figure 7:**
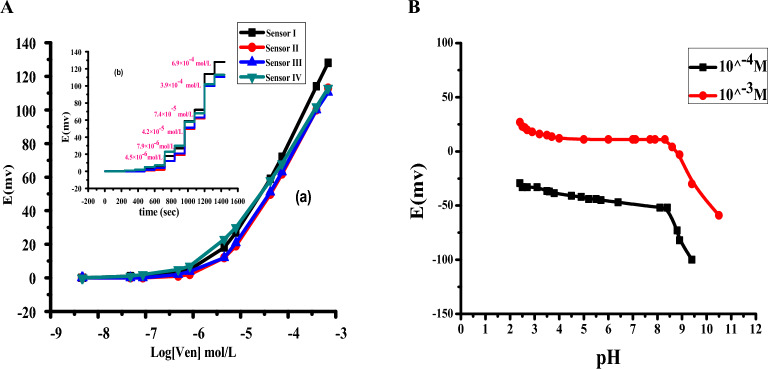
(**A**) Calibration plots (**a**) and time response (**b**) of sensor I (VEN-TPB−), sensor II (MWCNT/VEN-TPB−), sensor III (PANi/VEN-TPB−) and sensor IV (Ferrocene/VEN-TPB^−^) in phosphate buffer (0.01 mol/L) pH 6.0. (**B**) The effect of pH on the potentiometric response of the applied sensor (sensor II).

VEN-TPB^−^ ion-pair was used as an active sensing ionophore. This ion pair has precise efficiency in the determination of VEN ions in their solutions, as can be seen in Fig. 7Aa. The performance characteristics of the proposed sensor compared to three types of solid contact ISEs. These types were implemented with solid contact materials that were used as transducers of ion-to-electron, such as MWCNT, PANi, and ferrocene. These sensors were identified as C/VEN-ISE (sensor I), C/MWCNTs /VEN -ISEs (sensor II), C/PANi/VEN-ISEs (sensor III), and C/ferrocene/ VEN-ISEs (sensor IV). The analytical characteristics of the presented sensors are shown in Table 2. In Fig. 7Aa, the best analytical features raised from sensor II of MWCNT SC exhibited a cationic near–Nernestian slope of 56.1 ± 0.8 mV/decade with a detection limit of 3.8 × 10^–6^ mol/L (r^2^ = 0.999). However, there is no significant difference between the four proposed sensors in their characteristics features that confirm that introducing these types of ion-to-electron transducers into ISEs doesn't have a significant effect on their analytical features and potentiometric measurements. The potentiometric response of all proposed sensors was applied over the concentration range1.0 × 10^−7^– 1.0 × 10^−2^ mol /L of VEN solution. The potential measurements were detected every 5 s over 2 min for 1.0 mL added (0.5 by 0.5 mL) of each concentration. As shown in Fig. 7Ab, the applied sensors revealed fast response time (< 10 s), high accuracy (99.5–100.4%), and precision (0.3–1.4%) were distinguished.

**Table 2:** The analytical features and the potentiometric response of the proposed sensors.

Parameters	Sensor I	Sensor II	Sensor III	Sensor IV
Slope(mv/decade)	56.9 ± 1.2	56.1 ± 0.8	51.3 ± 1.3	46.4 ± 1.5
Detection limit, (mol /L)	3.9 × 10^–6^	3.8 × 10^–6^	3.5 × 10^–6^	3.1 × 10^–6^
Correlation coefficient (r^2^)	0.999	0.999	0.999	0.999
Linear range, (mol /L)	6.3 × 0^–6^–1.0 × 0^–2^	4.7 × 0^–6^–1.0 × 0^–2^	5.7 × 0^–6^–1.0 × 0^–2^	5.5 × 0^–6^–1.0 × 0^–2^
Response time, (s)	< 10	< 10	< 10	< 10
pH range	3.5–8.5	3.5–8.5	3.5–8.5	3.5–8.5
Precision, (%)	0.4	0.8	1.4	0.3
Accuracy, (%)	100.2	99.5	100.4	99.8
Standard deviation, (mV)	± 2.2	± 2.4	± 1.4	± 2.1

#### Method validation

##### Reproducibility and repeatability

The repeatability of the potentiometric response of the applied sensors was also studied. The test was carried out over 2.5 h by immersing the proposed sensor in 10 mL of 10 mmol/L of phosphate buffer (pH 6) and then was immersed in 10 mL of 10^–4^ mol/L of VEN for 30 min, alternately. The potential values were recorded, which revealed high stability and constant readings over the proceeding time. Also, it was an efficient and sophisticated sensor in the presence of transducers (MWCNT, PANi, and ferrocene). The reproducibility of the applied sensors proceeded over 8 weeks (n = 6). After 2 months, gradual changes in the detection limits and slopes were observed.

##### Ruggedness and robustness

The potentiometric characteristics of the applied sensor were studied over a wide range of variable pHs (2–10). The test was applied by using two concentrations of VEN (10^–3^ and 10^–4^ mol/L) with changes in the pH values that were adjusted with small aliquots of HCl and /or NaOH. Since the pKa of VEN is 9.4, VEN will be in the cationic at pH below 9.4. The effect of various pH levels on the properties of the utilized sensor was examined, and it was found that the sensor exhibited stability within a pH range of 3.5 to 8.5. However, at pH levels exceeding 8.5, the potential readings began to decrease due to the formation of non-ionized VEN. The wide range of stable potential measurements proved the robustness and durability of the potentiometric sensor under investigation (Fig. 7B). A working pH of 6 was chosen using a phosphate buffer solution with a concentration of 10 mmol/L to facilitate further potentiometric studies with the sensor.

### Sensors’ selectivity

The selectivity coefficient (log ${K}_{VEN,J}^{pot}$) determines the ability of an ISE to respond to an analyte *I* against an interfering ion *j*. The test was conducted to assess the influence of the different solid contact materials on the sensor's selectivity. Various interfering species were introduced, as different matrices can potentially be contaminated with VEN in their pharmaceutical forms or biological fluids. These interfering species included cationic species such as Na^+^, K^+^, Mg^2+^, and Ca^2+^ salts. Additionally, amino acids (valine, leucine, and glycine), sugars (fructose and lactose), and caffeine were added.

Table 3 presents the calculated log K ^pot^_VEN, J_, which indicated that the contaminated interfering ions did not significantly interfere with the determination of VEN. Furthermore, the transducers did not affect the selectivity of the proposed sensor for the VEN-TPB^−^ ion pair. The applied sensors verified high selectivity and efficacy in determining VEN in its solutions.

**Table 3:** The selectivity coefficients (log K ^pot^_VEN, J_ $$)$$ of the proposed sensor.

Interfering ion	- log K ^pot^_VEN, J_ ± SD			
	Sensor I	Sensor II	Sensor III	Sensor IV
K^+^	4.3 ± 0.1	4.8 ± 0.4	4.5 ± 0.3	4.5 ± 0.2
Na^+^	3.8 ± 0.4	4.8 ± 0.1	4.3 ± 0.2	4.0 ± 0.3
Mg^2+^	4.3 ± 0.3	4.8 ± 0.4	4.5 ± 0.2	4.5 ± 0.2
Ca^2+^	4.2 ± 0.1	4.8 ± 0.3	4.5 ± 0.4	4.5 ± 0.4
Valine	5.2 ± 0.5	4.8 ± 0.4	4.5 ± 0.1	4.5 ± 0.2
Leucine	4.1 ± 0.5	5.8 ± 0.2	5.4 ± 0.1	4.5 ± 0.3
Glycine	4.3 ± 0.4	4.8 ± 0.2	4.4 ± 0.3	4.5 ± 0.3
Caffeine	4.3 ± 0.3	3.8 ± 0.3	4.4 ± 0.3	4.5 ± 0.2
Fructose	4.3 ± 0.2	4.2 ± 0.2	4.5 ± 0.2	4.5 ± 0.1
Lactose	4.4 ± 0.2	4.2 ± 0.4	4.5 ± 0.1	4.5 ± 0.2

*SD (standard deviation) for n = 3.

### Analytical applications

#### Determination of VEN in pharmaceutical formulations

To evaluate the applicability of the sensor, modified with MWCNT, the VEN was detected in pharmaceutical tablets and synthetic urine samples as different matrices for investigating the electrode behavior.

From Table 4, the recoveries of VEN measurements were ranged (99.3–102.0%) comparing to the reference method of VEN determination using HPLC method^26^. F-test and t-test analyses were conducted to compare the two methods, and the results indicated no significant difference. This finding confirms the validity and reliability of the proposed sensor for accurately determining VEN in its solution.

**Table 4:** VEN determination in pharmaceutical preparations using the proposed sensor and reference method.

Pharmaceutical product and source	Nominal content taken, mg capsule^−1^	Found, mg capsule^−1^				F-test	t-Student test
		Proposed method	Recovery% ± SD	Reference method^26^	Recovery% ± SD		
Effexor XR Capsules, Wyeth, United Kingdom	75	75.3	100.4 ± 0.4	75.1	100.1 ± 0.6	2.2	1.6
	150	148.9	99.3 ± 0.6	149.5	99.7 ± 1.7	9.3	0.3
Venlafaxine Hydrochloride ER Capsules, Auro Pharma Inc, Canada	75	76.5	102.0 ± 1.4	74.2	98.9 ± 0.9	2.6	3.6

^a^Mean of three replicate measurements ± standard deviation (SD).

^b^F and t-Student tests at 95% confidence level values are 19.00 and 4.3, respectively.

#### Determination of VEN in spiked synthetic urine samples

Sensor-based MWCNT has been utilized to identify overdose patients, especially in circumstances requiring a prompt and precise evaluation diagnosis. Also, to study its selectivity in complex matrices.

Potentiometric measurements were applied after adding known VEN concentrations to synthetic urine samples using the recommended sensor. Table 5 illustrates the average recovery of 98.5 ± 0.4% of the spiked VEN concentration without any significant interference from the species commonly seen in artificial urine samples. This demonstrated that the recommended sensor might be utilized to determine the VEN content in complex matrices with little to no interference from their species.

**Table 5:** Recovery percents for determination of VEN in spiked synthetic urine samples.

Sample	VEN content (µM)		Recovery, (%)
	Spiked	Found	
1	5.0	4.94 ± 0.2	98.8 ± 0.3
2	10.0	9.59 ± 0.6	95.9 ± 0.2
3	15.0	14.61 ± 0.4	97.4 ± 0.4
4	20.0	20.42 ± 0.1	102.1 ± 0.7

## Conclusion

The novel potentiometric method for VEN determination, employing a solid contact screen-printed electrode (SPE), is a highly advanced, miniaturized, and reliable technique. The VEN-TPB^−^ ion-pair, used as the electroactive substance, displayed exceptional sensitivity and selectivity. The transduction mechanisms employed by the sensors differed, with the MWCNT sensor relying on double-layer formation, while the PANi and ferrocene sensors involved redox reactions. Among the tested sensors, the MWCNT-based sensor exhibited the most favorable outcomes, with a near-Nernstian slope of 56.1 ± 0.8 mV/decade, a detection limit of 3.8 × 10^–6^ mol/L (r^2^ = 0.999), and a low potential drift (∆E/∆t) of 34.6 µV/s. The MWCNT sensor also demonstrated the highest double-layer capacitance of 16.3 µF, and the specific capacitance of 0.0259 that sharply elevated, which it may be due to an electrical double-layer capacitor. The resistance values (R_b_) were determined as 0.4, 0.08, 0.3, and 0.3 MΩ for sensors I, II, III, and IV, respectively, with the MWCNT sensor exhibiting the lowest resistance. Results from electrochemical impedance spectroscopy (EIS), chronopotentiometry (CP) and cyclic voltammetry (CV) analyses further confirmed the superior performance, simplicity, accuracy, and durability of the MWCNT sensor.

## Data Availability

The data that support the findings of this study are available from the corresponding author on request.
